# The Perilous Journey of Patients With Primary CNS‐HLH to Bone Marrow Transplant: A Case Report

**DOI:** 10.1155/crh/1557951

**Published:** 2026-07-14

**Authors:** Camille Henyan, Braden Lane Garcia, Sarah Madison, Megan Gibbs, Maranda Diaz, Anish Ray

**Affiliations:** ^1^ Research Administration Office, Cook Children’s Medical Center, 801 Seventh Ave, Fort Worth, 76104, Texas, USA; ^2^ College of Biomedical and Translational Sciences, University of North Texas Health, 3500 Bowie Blvd, Fort Worth, 76107, Texas, USA

**Keywords:** central nervous system HLH (CNS-HLH), cytokine storm, hemophagocytic lymphohistiocytosis (HLH), neopterin, PRF1 mutation

## Abstract

Hemophagocytic lymphohistiocytosis (HLH) is a rare but life‐threatening syndrome of immune dysregulation with an incidence of 1.2–1.5 cases per million children annually. Curative therapy for primary HLH requires hematopoietic stem cell transplantation (HSCT). A subset of patients develop central nervous system (CNS) involvement, which is associated with high morbidity and mortality yet remains poorly characterized. We describe two patients with genetically confirmed primary HLH (PRF1 mutations with impaired perforin activity) who developed CNS disease. Both presented to the emergency department with fever. Common features included pancytopenia, hepatosplenomegaly, cerebrospinal fluid pleocytosis, and absence of malignancy on bone marrow biopsy. Patient 1: Presented with fever, irritability, abnormal gait, abnormal eye movements, and significant abnormal MRI findings. Patient 2: Following RSV and streptococcal pharyngitis, developed fever, poor oral intake, and lethargy. Both patients received HLH‐directed therapy (dexamethasone, etoposide, and emapalumab) plus intensive supportive care. Due to elevated neopterin and CNS disease, both underwent intrathecal chemotherapy to specifically target neuroinflammation. Outcomes diverged: Patient 1 developed refractory status epilepticus and died without transplantation, while Patient 2 successfully underwent HSCT and was discharged home. This case report highlights the heterogeneity of CNS involvement in primary HLH and its impact on outcomes. Elevated neopterin and neurologic manifestations may serve as critical early indicators of CNS disease. Despite advances in management strategies, timely recognition, aggressive treatment, and access to HSCT remain essential to survival. Broader clinical experience and collaborative studies are needed to optimize care strategies for CNS‐HLH.

## 1. Introduction

Hemophagocytic lymphohistiocytosis (HLH) is a rare hyperinflammatory syndrome characterized by excessive immune activation and a cytokine storm [1]. HLH presents in two forms. Primary (familial) HLH arises from autosomal recessive genetic mutations affecting cytotoxic pathways, while secondary HLH develops as an exaggerated immune response to infections, malignancies, autoimmune diseases, or metabolic disorders [1]. In a subset of patients, HLH involves the central nervous system (CNS), resulting in CNS‐HLH.

CNS‐HLH is not a distinct disease but a severe manifestation of systemic HLH. Although no standardized definition exists, CNS‐HLH is typically characterized by a triad of neurological symptoms, neuroimaging abnormalities, and cerebrospinal fluid (CSF) changes [2, 3]. Reported CNS involvement in HLH varies widely, occurring in approximately 33%–73% of patients [4, 5]. Common neurological features include seizures, altered consciousness, encephalopathy, meningism, ataxia, cranial neuropathies, and hemiparesis [3, 6]. MRI findings often demonstrate multifocal, bilateral T2‐weighted hyperintensities, confluent lesions, hemorrhage, atrophy, and calcifications, and CT scans may be useful when MRI is unavailable [3]. CSF findings may show hemophagocytosis, pleocytosis, and elevated protein levels [2].

Diagnosis of CNS‐HLH requires prior confirmation of HLH using the HLH‐2004 diagnostic criteria, which include either (1) a positive molecular diagnosis confirming primary HLH or (2) fulfillment of at least five of the following eight criteria [7]:

 Persistent fever
 Splenomegaly
 Cytopenias affecting at least two of three peripheral lineages
 Hypertriglyceridemia and/or hypofibrinogenemia
 Hemophagocytosis in bone marrow, spleen, or lymph nodes
 Low or absent NK cell activity
 Ferritin ≥ 500 µg/L
 Elevated soluble interleukin‐2 receptor (sIL‐2R) ≥ 2400 U/mL

In current practice, meeting 5/8 of the 2004 criteria raises the suspicion of HLH but can also be attributed to other conditions such as malignancies, infections, and rheumatoid disorders. For a definitive diagnosis and primary HLH, genetic testing is relied upon [7].

A diagnosis of CNS‐HLH is made when an individual that meets HLH criteria also demonstrates at least one of the three CNS features: neurological symptoms, neuroimaging findings, or CSF abnormalities [8].

This case report describes two pediatric patients with primary HLH and CNS involvement, emphasizing the diagnostic and therapeutic challenges associated with this presentation. It further explores neopterin—a macrophage activation marker released in response to interferon‐γ [9]—as a potential biomarker for CNS‐HLH. There is currently, although limited, evidence that neopterin may be associated with CNS‐HLH [8]. Because neopterin poorly crosses cellular membranes, when its concentration in CSF increases, it reflects macrophage activation within the blood–brain barrier and resultant CNS inflammation [10]. This suggests that neopterin could serve as a more specific indicator of CNS‐HLH than currently utilized markers. One major limitation surrounding the use of neopterin involves the fact that it can only be tested at select laboratories, often with prolonged time to result of up to several weeks. Our case reports add to the limited evidence of the potential association of neopterin and CNS‐HLH.

At present, no standardized treatment protocol exists for CNS‐HLH, and available literature remains limited. Therapeutic approaches typically mirror systemic HLH regimens and are adjusted based on the extent of CNS involvement. Intrathecal therapy (e.g., dexamethasone or methotrexate) may be used for severe CNS disease, while systemic treatments such as corticosteroids, etoposide, cyclosporine, and targeted biologics such as emapalumab (anti–IFN‐γ) aim to suppress hyperinflammation [5, 7, 8]. Hematopoietic stem cell transplantation (HSCT) remains the only curative option for primary HLH and its CNS manifestations [8, 11]. The following two cases illustrate varying disease courses and outcomes, highlighting the importance of early recognition and individualized management in CNS‐HLH.

## 2. Case Presentation

### 2.1. Patient 1

A previously healthy 20‐month‐old girl presented to the emergency department (ED) with one month of intermittent fever, progressive unsteady gait, reluctance to walk, and irregular eye movements and two days of consistent fever, rhinorrhea, and congestion. Her recent history included sinus infection, hand‐foot‐and‐mouth disease, and viral gastroenteritis. Two weeks prior, she was treated with oral iron for mild anemia. Initial head CT was normal. MRI under sedation demonstrated leptomeningeal enhancement in the supratentorial and infratentorial compartments, concerning for meningitis. Lumbar puncture showed lymphocyte‐predominant pleocytosis and elevated protein, suggesting postinfectious cerebellar encephalitis. She was treated with ceftriaxone, cefotaxime, and a two‐day course of intravenous immunoglobulin (IVIG), with clinical improvement at discharge.

Three days postdischarge, she re‐presented with fever and worsening gait instability. Repeat MRI revealed persistent enhancement suggestive of meningoencephalitis; MRA was normal. Laboratory evaluation showed anemia, leukopenia, elevated protein, and rising ferritin. Bone marrow biopsy showed trilineage hematopoiesis in a hypercellular marrow without evidence of malignancy or hemophagocytosis. CT imaging identified hepatosplenomegaly and pulmonary nodules. Dexamethasone (10 mg/m^2^) was initiated on illness Day 14 and later increased to 20 mg/m^2^ for worsening cerebellar inflammation and tremors.

**FIGURE 1:**
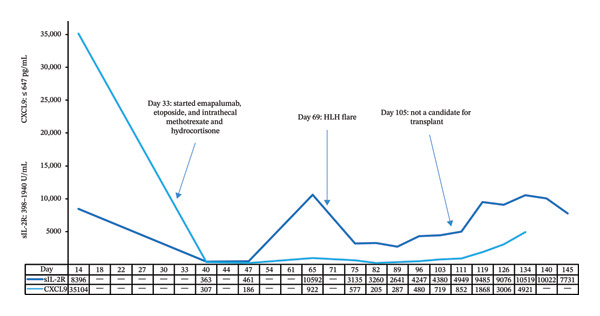
Trends in sIL‐2R and CXCL9 from first to last measurement for Patient 1. Note. Graphic representation of laboratory values soluble interleukin 2 receptor (dark blue line) and chemokine CXCL9 (light blue line).

Despite partial improvement, her soluble IL‐2R and CXCL9 levels rose sharply (8396 U/mL and 35,104 pg/mL, respectively; see Figure 1). On Day 27, she developed lethargy, abnormal eye movements, and seizures. EEG demonstrated right‐hemisphere status epilepticus, and MRI showed progression of cerebellar inflammation. She was diagnosed with HLH based on fever, cytopenia, hyperferritinemia, splenomegaly, hypofibrinogenemia, elevated cytokines, reduced perforin activity, and two PRF1 gene variants (c.445G > A, c.442G > A). Neopterin was elevated at over 500 nmol/L (reference range 7–65 nmol/L). Treatment included dexamethasone, twice‐weekly etoposide, weekly intrathecal methotrexate with hydrocortisone, and emapalumab (Gamifant).

Her hospital course was complicated by refractory seizures, respiratory failure, and prolonged intubation. Serial MRIs revealed progressive cerebral edema and inflammation. Despite transient laboratory improvement, she experienced an HLH flare with persistent neurologic deterioration. Due to severe neurological injury, she was not a candidate for stem cell transplantation, and immunosuppression was gradually tapered. She was discharged on Day 107 but continued to experience seizures, progressive encephalopathy, and systemic decline. She passed away at home on Day 181.

### 2.2. Patient 2

A previously healthy 11‐month‐old girl presented to the ED with one month of intermittent fever and recent evaluation for urinary tract infection. She had been treated unsuccessfully for RSV and streptococcal pharyngitis. Her mother reported daily fevers up to 103°F and decreased activity. Laboratory evaluation revealed pancytopenia, elevated CRP and CMP, hyponatremia, and hypoalbuminemia. Abdominal ultrasound demonstrated splenomegaly. She received cefepime, platelet and red blood cell transfusions, and was transferred to Cook Children’s Medical Center for the management of anemia, pancytopenia, and fever.

**FIGURE 2:**
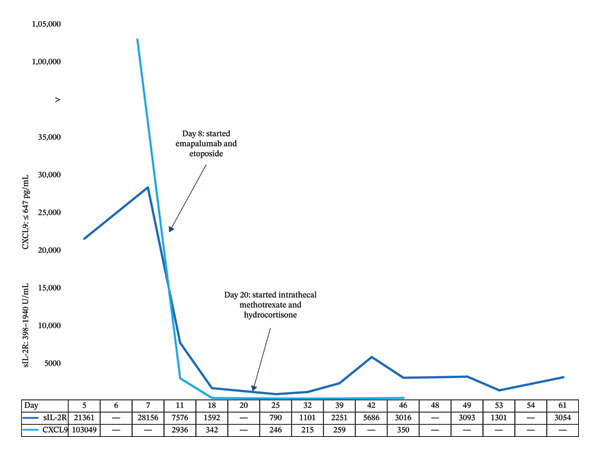
Trends in sIL‐2R and CXCL9 from first to last measurement for Patient 2. Note. Graphic representation of laboratory values soluble interleukin 2 receptor (dark blue line) and chemokine CXCL9 (light blue line). Patient 2 received bone marrow transplant on Day 79, not pictured on figure.

In the ICU, she received transfusion support, albumin, and diuretics for fluid overload. Bone marrow biopsy showed no evidence of malignancy or hemophagocytosis. CT imaging revealed splenic hypoechoic lesions. Ongoing fever and cytopenias contributed to meeting five of the eight HLH‐2004 criteria in addition to splenomegaly, hypofibrinogenemia, and hyperferritinemia. Lumbar puncture showed pleocytosis. PET scan revealed increased splenic uptake without malignancy. MRI brain demonstrated global parenchymal volume loss with ventricular dilation, highlighting ongoing neuroinflammation. HLH studies showed markedly elevated sIL‐2R and CXCL9 (21,361 U/mL and 103,049 pg/mL; see Figure 2), low perforin activity, and high CSF levels of IL‐8, IL‐10, and interferon‐γ. She was started on emapalumab, etoposide, and high‐dose steroids.

Hypertension developed during the second treatment week and was managed with amlodipine and intermittent labetalol. Genetic testing confirmed two PRF1 mutations (c.445G > A and c.50del). Elevated neopterin at 399 nmol/L (reference range 7–65 nmol/L) prompted initiation of intrathecal dexamethasone and methotrexate, resulting in improved alertness and stabilization of HLH markers. Cytopenias recovered gradually.

On Day 79, she underwent haploidentical HSCT from her father. Posttransplant management included cyclophosphamide, mycophenolate mofetil, and tacrolimus for GVHD prophylaxis. Early complications included gastrointestinal bleeding and hypertension requiring multiple antihypertensives. She later developed adenoviremia, treated with weekly IVIG. She was discharged after hematologic recovery and remained in remission at last follow‐up.

## 3. Discussion

These case reports highlight the diverse clinical manifestations and outcomes of primary HLH with CNS involvement. Both patients exhibited hallmark systemic features of HLH, alongside neurological and CSF abnormalities confirming CNS participation [7, 8]. Despite similar genetic profiles, clinical trajectories diverged, underscoring disease heterogeneity and the prognostic importance of early recognition and therapeutic escalation.

Neopterin, secreted by activated macrophages in response to interferon‐γ, demonstrated marked elevation in both patients reflecting their significant CNS involvement and supporting its potential as a biomarker for neuroinflammatory disease activity in HLH. Neopterin was more significantly elevated in Patient 1 who demonstrated more severe neuroinflammation and uncontrolled symptoms leading to her death. Because of its limited membrane permeability, elevated CSF neopterin may reflect intrathecal immune activation more specifically than other cytokine markers, offering diagnostic and prognostic value [10, 12].

Standardized management for CNS‐HLH is lacking, and approaches remain guided by HLH‐94 and HLH‐2004 protocols, supplemented with targeted CNS therapy. Intrathecal corticosteroids and methotrexate remain mainstays for acute management, often combined with systemic etoposide, dexamethasone, cyclosporine, or biologics such as emapalumab or alemtuzumab. HSCT remains the only curative therapy for primary forms, emphasizing the urgency of early systemic control to achieve transplant candidacy [11]. The urgency and clinical importance of achieving transplant candidacy is highlighted by Patient 1 passing without transplant and Patient 2 recovering posttransplant.

Overall, these two cases underscore key clinical lessons: CNS involvement in HLH can be both devastating and reversible; elevated CSF neopterin may serve as an early indicator; and successful outcomes depend on timely diagnosis, combined systemic and intrathecal therapy, and access to definitive transplantation. Our suspicions that neopterin may serve as an early indicator of CNS‐HLH need to be investigated with broader multicenter research studies with sample sizes sufficient for statistical testing. More generally, there is a need to refine diagnostic criteria, validate other biomarkers, and optimize neuroprotective treatment pathways for pediatric CNS‐HLH.

## Author Contributions

Camille Henyan completed record review and manuscript drafting. Braden Lane Garcia contributed to manuscript drafting. Sarah Madison and Megan Gibbs contributed to manuscript drafting and editing. Maranda Diaz completed medical documentation in an electronic medical system. Anish Ray contributed to project conception and manuscript editing. Anish Ray and Maranda Diaz were directly involved in patient care.

## Funding

Funding was provided by Cook Children’s Medical Center Research Administration Office. Camille Henyan and Braden Lane Garcia are supported with student stipends provided by the Cancer Prevention and Research Institute of Texas (grant number RP210046).

## Disclosure

All authors read, reviewed, and approved the final manuscript.

## Ethics Statement

Written informed consent was attained from parents of both patients. Case discussion includes age and gender with no other identifiable information. This study received an exemption from the Institutional Review Board at Cook Children’s Medical Center.

## Conflicts of Interest

Anish Ray is a speaker for Sobi biopharma company. The other authors declare no conflicts of interest.
